# The Nottingham recovery from COVID-19 research platform (NoRCoRP): Functional, clinical and patient-reported outcomes in adults referred to a post-COVID respiratory service

**DOI:** 10.1371/journal.pone.0344210

**Published:** 2026-03-05

**Authors:** Malik Hamrouni, Ayushman Gupta, Sophie Middleton, Sabrina Prosper, Theresa Harvey-Dunstan, Joanne Porte, Tricia M. McKeever, Ian P. Hall, Charlotte E. Bolton

**Affiliations:** 1 NIHR Nottingham Biomedical Research Centre, Nottingham, United Kingdom; 2 Centre for Respiratory Research, Translational Medical Sciences, School of Medicine, University of Nottingham, Nottingham, United Kingdom; 3 Respiratory Medicine, Nottingham University Hospitals Trust, Nottingham, United Kingdom; SMS Medical College and Hospital, INDIA

## Abstract

**Aims:**

To characterise symptoms, function and patient-reported outcome measures (PROMs), and identify associated factors in adults with persisting respiratory symptoms post-COVID.

**Methods:**

Cross-sectional analysis of 210 non-hospitalised adults referred to a post-COVID respiratory clinic (December 2020-July 2024) who consented to research. Assessments included demographics, symptoms, lung function, chest CT, and several PROMs: MRC dyspnoea score, Nijmegen Questionnaire score (NQ), Hospital Anxiety and Depression Scale, Chalder Fatigue Scale, Short Physical Performance Battery (SPPB) and Fried Frailty Index. Multivariate logistic regression examined key exposure-outcome associations.

**Results:**

Among participants (mean age 49.4 years; 68% female; median 13.3 months since COVID-19 diagnosis), 95% reported shortness of breath, 54% had clinically significant breathlessness (MRC ≥ 3), 68% had an NQ score (>23) consistent with dysregulated breathing, 32% had a low SPPB score (<10), and 77% were classed as frail/pre-frail, despite the majority being of working age. Nearly half (47%) of those employed pre-infection had not returned to previous hours. Spirometry and CT abnormalities were not common. Higher body mass index (odds ratio = 1.10, 95% confidence interval = 1.05–1.16, n in model = 190) and depression (2.25, 1.13–4.56, n = 164) were associated with MRC ≥ 3. Dysregulated breathing was associated with female sex (3.63, 1.77–7.60, n = 186), current/ex-smoker (2.56, 1.25–5.47, n = 186), fatigue (8.87, 2.59–37.0, n = 162), anxiety (3.57, 1.70–7.69, n = 162) and depression (5.70, 2.59–13.40, n = 162). A low SPPB score was associated with female sex, current smoking, depression, clinically significant breathlessness, dysregulated breathing, and greater deprivation.

**Conclusion:**

In non-hospitalised patients with persistent respiratory symptoms post-COVID, dysregulated breathing, deconditioning and psychological distress were key factors linked with symptom burden. These findings suggest a multidisciplinary approach should be considered to optimise recovery.

## Introduction

Although many individuals recover from acute COVID-19, a notable proportion experience persistent symptoms [1]. A meta-analysis including over 700 000 participants, with an average follow-up time of 4 months, found that almost half reported at least one unresolved symptom [2]. Such symptoms are heterogeneous and include fatigue, weakness, breathlessness, impaired sleep quality and cognitive difficulties [2,3].

Persisting respiratory symptoms are particularly common post-COVID with breathlessness being the most frequent [4], having a prevalence of 51% in the PHOSP-COVID cohort, even a year after hospital discharge [5]. While non-hospitalised individuals remain relatively under-studied, small cohort studies report impaired lung function, fatigue, reduced physical capacity and a high prevalence of dysfunctional breathing (following strong feedback from clinic attendees and the patient research panel, we will henceforth use the term dysregulated breathing) [6,7]. However, such studies are limited in size and scope and often do not offer comprehensive phenotyping.

Notably, many previously healthy and active individuals report breathlessness, despite normal spirometry or chest CT [8–10], yet no universal, standardised care pathway exists for such individuals. Although the aforementioned sequelae are described in the literature, less is known about factors associated with breathlessness, dysregulated breathing and poor physical function – particularly in non-hospitalised patients. Further, although randomised controlled studies report benefit of pulmonary rehabilitation [11], the breadth of symptoms and scale of those still affected is substantial, highlighting the need to identify who is most at risk and which modifiable factors should be prioritised to inform management and rehabilitation strategies.

The Nottingham Recovery from COVID-19 Research Platform (NoRCoRP) was established to profile individuals with persisting respiratory symptoms who had been referred into a diagnostic respiratory clinic and consented to be part of a research registry. Participants within the registry were extensively characterised – including standard clinical tests and symptoms alongside physical performance testing and an array of patient-reported outcomes. Using data from NoRCoRP, this study describes the clinical characteristics of patients not hospitalised during their acute infection and identifies factors associated with adverse respiratory health and functional limitation post-COVID.

## Materials and methods

### Study design and participants

This was a cross-sectional single-centre study based at Nottingham University Hospitals (NUH) NHS Trust. Adults (≥ 18 years) referred from primary care to a post-COVID respiratory clinic were eligible; only those not hospitalised during their acute COVID-19 illness were included in this analysis. Referrals were made for persistent respiratory symptoms and/or new imaging changes after COVID-19 and were triaged by a clinician within NUH respiratory services. The minimum symptom duration required for referral was ≥ 6 weeks. The clinic did not accept referrals based on non-respiratory symptoms in isolation (e.g., fatigue, myalgia, brain fog). Individuals with pre-existing respiratory disease were not excluded. During the study period, South Nottingham and City had no formal commissioned long COVID out-patient services for non-hospitalised patients to medically evaluate and assess presenting symptoms. The respiratory clinic at NUH provided a single, multidisciplinary team to assess patients, and avoid them being triaged across general respiratory outpatient clinics at NUH. The NoRCoRP registry was established to capture a breadth of patient-reported outcome measures (PROMs) alongside the clinical assessment. Recruitment ran from December 11^th^ 2020 to July 19^th^ 2024 with consecutive patients attending being approached for NoRCoRP during this period (convenience sampling). Ethical approval was obtained from the London–Bromley Research Ethics Committee (20/HRA/3732). Consent was obtained either in clinic using a written informed consent form (ICF), or remotely via a verbal/electronic consent form (VECF) during a telephone call with the study team (to allow additional time for consideration and minimise face-to-face contact during the COVID-19 pandemic). For the VECF pathway, verbal consent to contact was first obtained and recorded in the medical notes. Verbal consent followed an ethics committee–approved script, and a copy of the ICF or VECF was stored in the patient notes. This study is reported in line with STROBE guidelines [12].

### Recruitment

Eligible participants were invited by a clinician to take part and sent a participant information sheet, usually before their face-to-face consultation. When in-person visits were either not required or declined by the participant, consent was obtained verbally over the telephone. For these participants, research questionnaires with a pre-paid return envelope were also mailed out.

### Sociodemographics

Age, sex, ethnicity (white, non-white), smoking status (never, former, current), COVID-19 vaccination status, Index of Multiple Deprivation (IMD) quintile (from postcode), and pre-COVID employment and back-to-work status were recorded.

### Clinical measures

Pre-existing lung disease was documented at the clinic visit. Height and weight were recorded and used to calculate body mass index (BMI). Resting peripheral oxygen saturation (SpO_2_) on room air and resting heart rate were recorded using a pulse oximeter. As part of routine care, participants underwent blood tests within ± 6 weeks of the clinic visit and/or during the visit, including C-reactive protein (CRP), full blood count (with eosinophil count) and B-type natriuretic peptide (BNP), where clinically indicated. Spirometry and chest computed tomography (CT) were also conducted where clinically indicated. Spirometry was performed by the NUH Trust lung physiology staff during the clinic visit: forced expiratory volume in one second (FEV_1_) and forced vital capacity (FVC), with predicted values based on the Global Lung Function Initiative (GLI-2012) [13]. Airflow obstruction was defined as an FEV_1_/FVC ratio < 0.7. A restrictive spirometry pattern was defined as a preserved FEV1/FVC ratio (≥0.7) with both FEV1 and FVC < 80% predicted. Chest CT scans (requested more frequently early in the pandemic when post-COVID sequelae were less understood) were categorised by presence or absence of parenchymal abnormality: bronchiectasis, atelectasis, emphysema, post-COVID fibrosis, other fibrotic changes, other interstitial abnormalities.

### Physical performance and frailty assessments

The Short Physical Performance Battery (SPPB) [14] and the Fried frailty index [15], following standard protocols were used. Assessments were conducted by either a specialist respiratory physiotherapist, or a senior respiratory research practitioner who was trained by the former. A score <10 on the SPPB was classed as a low score indicative of functional limitation [16]. The Fried Frailty Index gives individuals a score based on how many of the following criteria are met: unintentional weight loss in the last year, self-reported exhaustion, weakness (grip strength), slow walking speed and low physical activity. Individuals are then classed as frail (score ≥3), pre-frail (1–2) and not frail (0). Further details on the scoring system can be found elsewhere [15].

### Patient-Reported Outcome Measures (PROMs)

The following validated PROMs were used with the following cut-offs: MRC breathlessness scale (scores ≥3 classed as clinically significant breathlessness) [17], the Nijmegen Questionnaire (scores >23 classed as dysregulated breathing for study analyses [18]; however, in clinical care, diagnosis was made after assessment by a clinician); Chalder Fatigue Scale (bimodal scoring, with scores ≥4 suggestive of fatigue) [19]; the Hospital Anxiety and Depression Scale (HADS) [20], with scores ≥8 in their respective domains indicating anxiety or depression [21], and the EuroQol Visual Analogue Scale (EQ-VAS) from the EQ-5D questionnaire [22].

### Statistical analysis

Descriptive statistics for participant demographics were presented and mean and standard deviation for continuous variables and number and percentage for categorical variables. Logistic regression was used to assess the associations of exposure variables (all sociodemographic variables with the exception of employment/back-to-work status due to limited data, Chalder Fatigue Scale score, and HADS anxiety and depression domain score) with clinically significant breathlessness, dysregulated breathing and functional limitation. Airflow obstruction was treated as an additional exposure for clinically significant breathlessness, and presence/absence of clinically significant breathlessness and dysregulated breathing were also treated as exposure variables for functional limitation. Given conceptual overlap and expected correlation between PROMs, they were not simultaneously included in the same regression models *a priori* to avoid multicollinearity and over-adjustment. A correlation heatmap was created to show the strength of relationship between PROMs. To examine the impact of dysregulated breathing and functional limitation on EQ-5D VAS, linear regressions and ANCOVAs were performed. Two sets of sensitivity analyses were conducted. First, to assess potential effect modification by time since infection, this was coded as a binary variable (<12 vs ≥ 12 months) and confounder/exposure-by-time interaction terms were fitted for each predictor-outcome association in unadjusted models. Second, any individuals with airflow obstruction or fibrosis were excluded and exposure-outcome associations were re-assessed. Adjusted models include all sociodemographic variables as covariates. For all regression models, variance inflation factor was checked to be below 2.5 to minimise risk of multicollinearity. A complete case approach was used for each outcome. Details on the handling of missing data and patterns of missingness are reported in S1 Text. Statistical significance was accepted at p < 0.05 (i.e., 95% confidence intervals not crossing 1). All statistical analyses were performed using **R** version 4.5.1 (R Core Team, Vienna, Austria). The R packages used in regression analyses are listed in S2 Text.

## Results

### Demographics

Of the 249 people who attended the clinic, 39 were ineligible (hospitalised during their initial COVID infection and/or did not have at least one respiratory symptom), leaving a final analytical sample of 210 participants. A participant flow chart can be found in S1 Fig. Participants had a mean (standard deviation = SD) age of 49.4 (12.9) years, and 142 (68%) were female. Complete participant demographics are presented in Table 1. The vast majority of participants (88%) were <65 years old, of working age. Of those with available occupation data who were employed prior to their acute infection (n = 146/173, 84%), at the clinic visit, only 78/146 (53%) had returned to their previous hours, 34 (23%) had returned but worked fewer hours and 34 (23%) had not returned. The median time between COVID-19 diagnosis and the clinic visit was 13.3 months (interquartile range = 10.1–18.9) with a minimum and maximum of 4.1 and 43.5 months, respectively. Almost all (97%) had received at least two COVID-19 vaccinations by the time of the consultation.

**Table 1:** Participant demographics as mean (SD) or N (%).

Variable	Value
Age (years) (n = 210)	49.4 (12.9)
***Sex* (n = 210)**	
Male	68 (31%)
Female	142 (68%)
**BMI (kg/m** ^**2**^ **) (n = 194)**	31.2 (8.2)
***Ethnicity* (n = 207)**	
White	182 (88%)
Non-white	25 (12%)
***Smoking status* (n = 207)**	
Never	121 (59%)
Former	68 (33%)
Current	18 (9%)
***IMD quintile* (n = 205)**	
1 (most deprived)	38 (19%)
2	34 (17%)
3	41 (20%)
4	39 (19%)
5 (least deprived)	53 (26%)

BMI, body mass index; IMD, index of multiple deprivation

## Clinical characteristics

### Symptoms and vital signs at the time of clinic visit and pre-COVID respiratory disease

The most common symptoms at presentation to clinic were shortness of breath (n = 199, 95%), dry cough (n = 112, 53%) and productive cough (n = 53, 25%). Other frequently reported non-respiratory symptoms were fatigue (n = 180, 86%), low mood (n = 133, 64%), muscle aches (n = 129, 62%), anxiety (n = 125, 60%), and malaise (n = 113, 54%). Almost all participants (98%) reported more than one symptom. The patients with available data (n = 194) had a mean resting blood oxygen saturation level of 97.4% (1.6) while breathing room air. No participants had a saturation level <92% at rest. Mean resting heart rate was 84 (14) bpm. Pre-COVID respiratory diagnoses were reported as follows: asthma 39/206 (19%), COPD 3/206 (1%), and bronchiectasis 1/206 (<1%).

### Blood test results

There were 20/93 (22%) patients with an eosinophil count >0.3 × 10^9 cells/L; 10/77 (13%) had a CRP concentration ≥10 mg/L, 21 (27%) were between ≥5 and <10 mg/L, and the remaining 46 (60%) were <5 mg/L. Of the 72 with BNP readings, 67 (93%) were ≤100 (ng/L), and the remaining five (7%) ranged between 121–204 (ng/L).

### Spirometry at clinic visit

Among the 188 patients who underwent spirometry, the mean (SD) % predicted FEV1 and FVC values were 95% (19) and 100% (17), respectively. Airflow obstruction was evident in 23/188 (12%) of patients; of these, 9/188 had an FEV₁ ≥ 80% predicted, 11/188 had 50–79% predicted, and 3/188 had 30–49% predicted. Eighteen of 188 participants (10%) had a restrictive spirometry picture.

### CT scan results

Chest CT scans were performed in 146/210 (70%) participants. Among those scanned, bronchiectatic changes were identified in 7/146 (5%), post-COVID fibrosis in 4/146 (3%), other fibrotic changes in 3/146 (3%), other interstitial abnormalities in 13/146 (9%), atelectasis in 31/146 (22%) and emphysema in 7/146 (5%), most of which were of little clinical relevance.

### Physical performance and frailty assessments

Almost a third (55/170, 32%) had a low SPPB score. A fifth (34/169, 20%) were classed as frail and 57% as pre-frail (97/169). The number of people meeting each criterion for the Fried Frailty Index were as follows: weakness (23%), slow walking speed (41%), low physical activity (20%), unintentional weight loss (12%), and exhaustion (54%). The respective mean (SD) ages of those with a low SPPB score and those with frailty were 49.4 (12.4) and 46.2 (13.3) years, similar to the mean age of the entire study population.

### PROMs

According to the MRC dyspnoea scale, 113/210 (54%) reported clinically significant breathlessness. Using pre-specified cut-off points, the Nijmegen questionnaire revealed that 133/195 (68%) had elevated scores. The Chalder Fatigue total score was elevated in 160/176 (91%). The HADS anxiety and depression domain scores suggest that 112/176 (64%) and 93/176 (53%) had symptoms of anxiety and depression respectively. Pairwise overlap of these outcomes in addition to SPPB score is shown in S2 Table, and an UpSet plot to visualise all possible combinations can be found in S2 Fig. A correlation heatmap is presented in S3 Fig, which shows that all PROMs exhibited statistically significant correlations with each other (all p ≤ 0.01), except for the MRC dyspnoea scale and SPPB, which did not exhibit statistically significant correlations with the HADS anxiety score. Mean (SD) EQ-VAS score was 56.6 (19.4) in the 174 individuals with available data.

### Factors associated with clinically significant breathlessness (MRC dyspnoea score ≥3)

The adjusted models revealed that BMI and depression were associated with higher odds of clinically significant breathlessness (Table 2).

**Table 2:** Factors associated with an MRC dyspnoea score ≥3.

Variable	N for model	Unadjusted OR (95% CI)	Unadjusted p-value	Adjusted OR (95% CI)	Adjusted p-value
Age (years)	190	1.01 (0.99 to 1.03)	.38	1.00 (0.98 to 1.03)	.80
***Sex***					
Male	190	1.00 (ref.)		1.00 (ref.)	
Female		1.34 (0.73 to 2.48)	.35	1.23 (0.62 to 2.47)	.55
BMI (kg/m^2^)	190	**1.11 (1.06 to 1.16)**	**<.001**	**1.10 (1.05 to 1.16)**	**<.001**
***Ethnicity***					
White	190	1.00 (ref.)		1.00 (ref.)	
Non-white		0.43 (0.17 to 1.04)	.06	0.49 (0.18 to 1.31)	0.16
***Smoking***					
Never smoked	190	1.00 (ref.)		1.00 (ref.)	
Ex-smoker		1.39 (0.74 to 2.61)	.31	1.21 (0.60 to 2.43)	.60
Current		1.21 (0.43 to 3.55)	.72	1.29 (0.42 to 4.16)	.66
***IMD quintile***					
5	190	1.00 (ref.)		1.00 (ref.)	
4		0.82 (0.34 to 1.97)	.66	0.73 (0.28 to 1.88)	.51
3		0.96 (0.40 to 2.32)	.93	0.99 (0.38 to 2.57)	.98
2		0.62 (0.25 to 1.51)	.29	0.58 (0.22 to 1.54)	.28
1		1.03 (0.43 to 2.52)	.95	0.71 (0.27 to 1.87)	.49
***Airflow obstruction***					
No	170	1.00 (ref.)		1.00 (ref.)	
Yes		0.94 (0.37 to 2.46)	.90	1.15 (0.39 to 3.53)	.80
***Chalder Fatigue Scale***					
No fatigue	164	1.00 (ref.)		1.00 (ref.)	
Fatigued		2.59 (0.91 to 7.96)	.08	1.81 (0.58 to 5.98)	.31
***HADS anxiety***					
No anxiety	164	1.00 (ref.)		1.00 (ref.)	
Anxiety		1.08 (0.57 to 2.05)	.82	1.01 (0.50 to 2.04)	.98
***HADS depression***					
No depression	164	1.00 (ref.)		1.00 (ref.)	
Depression		**2.18 (1.16 to 4.14)**	**.016**	**2.25 (1.13 to 4.56)**	**.022**

Odds ratios for continuous variables (age and BMI) are per 1-unit increase.

BMI, body mass index; IMD, index of multiple deprivation; HADS, Hospital Anxiety and Depression Scale. Adjusted models include age, sex, ethnicity, smoking and IMD quintile as covariates (unless they are the exposure variable).

Significant associations (p < 0.05) are highlighted in bold.

### Factors associated with dysregulated breathing

Dysregulated breathing was markedly more common in women (103/131, 79%) than men (30/64, 47%), and in those with fatigue (109/150, 73%) than those without (4/16, 25%). Due to low numbers of current smokers who were not classed as having dysregulated breathing (n = 1), current and ex-smokers were combined for this analysis specifically. After adjustment, the odds of dysregulated breathing were higher in women, ex-smokers, and those with fatigue, anxiety and depression (Table 3).

**Table 3:** Factors associated with elevated Nijmegen score (dysregulated breathing).

Variable	N for model	Unadjusted OR (95% CI)	Unadjusted p-value	Adjusted OR (95% CI)	Adjusted p-value
Age (years)	186	**0.97 (0.95 to 1.00)**	**.031**	0.98 (0.95 to 1.01)	.18
***Sex***					
Male	186	1.00 (ref.)		1.00 (ref.)	
Female		**4.30 (2.22 to 8.48)**	**<.001**	**3.63 (1.77 to 7.60)**	**<.001**
BMI (kg/m^2^)	186	1.01 (0.98 to 1.06)	.47	1.01 (0.97 to 1.06)	.72
***Ethnicity***					
White	186	1.00 (ref.)		1.00 (ref.)	
Non-white		2.15 (0.76 to 7.71)	.19	2.29 (0.71 to 9.06)	.19
Smoking*					
Never smoked	186	1.00 (ref.)		1.00 (ref.)	
Current/Ex-smoker		**2.00 (1.05 to 3.93)**	**.038**	**2.56 (1.25 to 5.47)**	**.012**
***IMD quintile***					
5	186	1.00 (ref.)		1.00 (ref.)	
4		0.91 (0.37 to 2.29)	.85	0.92 (0.34 to 2.51)	.87
3		1.29 (0.50 to 3.41)	.60	1.19 (0.43 to 3.38)	.74
2		1.38 (0.53 to 3.76)	.52	1.17 (0.40 to 3.51)	.78
1		1.49 (0.57 to 4.05)	.42	1.29 (0.45 to 3.85)	.64
***Chalder Fatigue Scale***					
No fatigue	162	1.00 (ref.)		1.00 (ref.)	
Fatigued		**7.95 (2.60 to 29.80)**	**<.001**	**8.87 (2.59 to 37.0)**	**.001**
***HADS anxiety***					
No anxiety	162	1.00 (ref.)		1.00 (ref.)	
Anxiety		**3.64 (1.83 to 7.35)**	**<.001**	**3.57 (1.70 to 7.69)**	**<.001**
***HADS depression***					
No depression	162	1.00 (ref.)		1.00 (ref.)	
Depression		**4.94 (2.44 to 10.50)**	**<.001**	**5.70 (2.59 to 13.40)**	**<.001**

Odds ratios for continuous variables (age and BMI) are per 1-unit increase.

*Current and ex-smokers were combined due to low numbers

BMI, body mass index; IMD, index of multiple deprivation; HADS, Hospital Anxiety and Depression Scale.

Adjusted models include age, sex, ethnicity, smoking and IMD quintile as covariates (unless they are the exposure variable).

Significant associations (p < 0.05) are highlighted in bold.

### Factors associated with a low SPPB score

In adjusted models, female sex, current smoking, depression, clinically significant breathlessness, and dysregulated breathing were associated with higher odds of a low SPPB score. IMD quintiles 1, 2 and 3 were associated with at least fourfold higher odds compared to quintile 5 (Table 4). Due to low numbers of individuals who did not have fatigue and a low SPPB score, the association between these variables was not assessed.

**Table 4:** Factors associated with a low SPPB score (<10).

Variable	N for model	Unadjusted OR (95% CI)	Unadjusted p-value	Adjusted OR (95% CI)	Adjusted p-value
Age (years)	164	1.00 (0.98 to 1.03)	.91	1.02 (0.99 to 1.05)	.25
***Sex***					
Male	164	1.00 (ref.)		1.00 (ref.)	
Female		**2.61 (1.25 to 5.81)**	**0.014**	**3.14 (1.31 to 8.17)**	**.014**
BMI (kg/m^2^)	164	1.02 (0.98 to 1.06)	0.32	1.01 (0.97 to 1.06)	.70
***Ethnicity***					
White	164	1.00 (ref.)		1.00 (ref.)	
Non-white		1.42 (0.52 to 3.67)	.47	1.50 (0.49 to 4.45)	.47
***Smoking***					
Never smoked	164	1.00 (ref.)		1.00 (ref.)	
Ex-smoker		1.62 (0.80 to 3.28)	.18	1.88 (0.84 to 4.27)	.13
Current		3.16 (0.88 to 11.8)	.08	**4.63 (1.13 to 20.80)**	**.036**
***IMD quintile***					
5	164	1.00 (ref.)		1.00 (ref.)	
4		1.88 (0.56 to 6.51)	.31	2.50 (0.69 to 9.39)	.16
3		**7.49 (2.57 to 24.50)**	**<.001**	**9.32 (3.00 to 33.0)**	**<.001**
2		**3.89 (1.30 to 12.70)**	**0.018**	**4.10 (1.27 to 14.50)**	**.021**
1		**4.35 (1.44 to 14.40)**	**0.011**	**4.46 (1.38 to 15.80)**	**.015**
***HADS anxiety***					
No anxiety	144	1.00 (ref.)		1.00 (ref.)	
Anxiety		2.00 (0.96 to 4.36)	.07	1.45 (0.62 to 3.43)	.39
***HADS depression***					
No depression	144	1.00 (ref.)		1.00 (ref.)	
Depression		**2.76 (1.35 to 5.81)**	**0.006**	**2.83 (1.26 to 6.61)**	**.014**
***MRC dyspnoea score***					
1-2	164	1.00 (ref.)		1.00 (ref.)	
≥3		**2.02 (1.03 to 4.09)**	**0.044**	**2.50 (1.12 to 5.02)**	**.028**
***Nijmegen score***					
Normal	162	1.00 (ref.)		1.00 (ref.)	
Dysregulated		**3.44 (1.58 to 8.16)**	**0.003**	**2.49 (1.03 to 6.43)**	**.049**

BMI, body mass index; IMD, index of multiple deprivation; HADS, Hospital Anxiety and Depression Scale.

Adjusted models include age, sex, ethnicity, smoking and IMD quintile as covariates (unless they are the exposure variable). Significant associations (p < 0.05) are highlighted in bold.

### Association of Nijmegen and SPPB scores with health-related quality of life

ANCOVAs (adjusted for age, sex, BMI, ethnicity, smoking and IMD quintile) revealed that individuals with dysregulated breathing had a significantly worse EQ-5D VAS score than individuals with a normal Nijmegen score (56 vs 70, p < 0.001). Similarly, a low SPPB score was associated with a worse EQ-5D VAS score than a normal SPPB score (58 vs 68, p < 0.001). In multivariable linear regression with the same covariate adjustment and both predictors included as continuous variables, a 1-SD increase in Nijmegen score (SD = 11.1) was associated with a 7.85-point reduction in EQ-5D VAS (95% CI −11.1 to −4.62, p < 0.001), while a 1-SD increase in SPPB score (SD = 2.0) was associated with a 4.94-point increase in EQ-5D VAS (95% CI 1.66 to 8.23, p = 0.004). Nijmegen score and SPPB score explained 12.7% and 5.7% of the total variance in EQ-5D VAS, respectively.

### Sensitivity analysis

No significant interaction effects for time since infection with confounders/exposures were observed, indicating the associations were broadly consistent across follow-up durations. After removing individuals with fibrosis and airflow obstruction and retesting exposure-outcome associations, all previously significant associations remained with a few exceptions. The association of current smoking with dysregulated breathing was attenuated, as were the associations of breathlessness, dysregulated breathing and sex with a low SPPB score.

## Discussion

This study provides a detailed profile of non-hospitalised adults with persistent respiratory symptoms after COVID-19 infection. The majority of patients experienced clinically significant breathlessness and dysregulated breathing, which is not surprising given that respiratory symptoms were required for referral to the clinic and therefore for study inclusion. Additionally, a large proportion were frail and limited in their physical performance, which was unlikely to be explained by older age. The symptom burden is reflected by the alarming finding that only about half of those employed pre-infection were able to return to their previous hours after COVID-19. Importantly, several key factors associated with clinically significant breathlessness, dysregulated breathing and functional limitation were identified, extending the evidence base beyond the more-studied hospitalised patients and underscoring the detrimental impact of COVID-19 months to years after milder acute infection.

Spirometry and CT scan results were largely normal, with lower frequencies of radiological abnormalities than reported in the PHOSP-COVID cohort [23]. We therefore deem it unlikely that airflow obstruction and lung parenchymal abnormalities were key contributing factors to breathlessness in the cohort, echoing previous small cohort studies in non-hospitalised patients [6]. The current findings instead point to BMI as well as mood disturbance through amplifying symptom perception. Although previous studies have documented such associations, they were in patients hospitalised during the initial infection [24,25]. While altered respiratory mechanics due to adiposity could partly explain the association between BMI and breathlessness in theory, without pre-COVID data, temporality is uncertain and reverse causation cannot be ruled out. Indeed, persistent breathlessness after the acute illness may have led to reduced physical activity and subsequent weight gain. Additionally, residual confounding through undiagnosed pre-existing health conditions could also contribute to a relationship between BMI and breathlessness. Longitudinal research with measurement of physical activity and robust confounder adjustment is needed to better understand the independent contribution of BMI in post-COVID respiratory symptoms. Either way, these observations align with recent research priorities for breathlessness, including identifying non-drug treatments such as exercise/weight management, strategies that may improve symptom management, cost-effective assessment pathways when routine tests are unrevealing, and designing services that can provide sustained support for long-term wellbeing [11,26].

As per previous research, women and those with anxiety and depression were at higher odds of having dysregulated breathing [27,28]. Women may be at higher risk due to smaller lungs and narrower airways relative to lung size [29], which may make a given change in breathing capacity more noticeable, especially during exertion [30]. Fatigue was also associated with dysregulated breathing. This co-existence may not only reflect shared contributors – an interplay of deconditioning, reduced activity, heightened interoceptive sensitivity, and mood disturbance – driving habitual breathing pattern changes, but also a bi-directional relationship (fatigue promoting inefficient breathing and vice versa). Dysregulated breathing is likely amenable to breathing retraining, pulmonary rehabilitation, and psychological support. Additionally, the current findings suggest more rigorous screening is warranted in women, those reporting fatigue, and individuals with symptoms of anxiety and depression.

Almost a third of patients had functional limitation according to the SPPB, which is alarming for a largely working-age cohort. In line with previous studies in post-COVID populations, there was no association between age and functional limitation, suggesting the latter is not confined to older adults and may be driven more by post-COVID sequelae [31]. The degree of functional limitation in the present cohort likely hinders their ability to return to work, which in turn carries wider economic implications, and suggests occupational rehabilitation approaches may be warranted for such individuals [32]. Importantly, given evidence for distinct long COVID phenotypes (for example, predominant respiratory symptoms in the present sample), future research should determine whether symptom- and phenotype-specific factors can better inform the development of individualised return-to-work plans. Functional impairment was linked with clinically significant breathlessness, dysregulated breathing, and mood disorder, which may be indicative of a self-reinforcing cycle: breathlessness/dysregulated breathing reduce activity, facilitating deconditioning, which further compromises respiratory and physical function and amplifies symptom perception and may contribute to mental health issues. Again, a multi-disciplinary approach, with breathing exercises, pulmonary rehabilitation and psychological therapies may help to break such a cycle. Social determinants have previously been shown to have a marked impact on COVID-19 recovery [33]. The social gradient observed herein (higher odds of functional limitation in the three most deprived IMD quintiles) further suggests a risk of widening inequalities. Women were also much more likely to have functional limitation. Taken together, the current findings may help health services in identifying those who are most vulnerable to persisting COVID symptoms, a key research focus in this field [34]. Indeed, this will help inform equitable rehabilitation strategies and reduce apparent health inequalities. The findings also address key research questions on long-term COVID sequelae, raised through a joint patient and clinician priority setting exercise, by highlighting rehabilitation strategies to support recovery and reduce activity-limiting symptoms and functional impairment [35]. Additionally, these findings may inform current clinical pathways, by demonstrating the need for follow-up care even when airflow obstruction and/or structural lung abnormalities are not identified. In line with previous research, the absence of such findings does not preclude debilitating respiratory symptoms and/or functional limitation [31].

Health-related quality of life was lower in those with dysregulated breathing and poorer physical function, further stressing that controlled trials of breathing retraining and/or physical rehabilitation to improve long-term well-being are warranted. Longitudinal cohorts with sufficient follow-up may also help in determining whether improving these domains will indeed yield meaningful and sustained quality of life improvements.

A limitation of the present study is the absence of pre-COVID data which prevents precise quantification of the extent to which changes are attributable to the acute infection and its recovery. The study being cross-sectional prevents us from establishing causality and measuring incidence. Also, given that certain associations may be bi-directional, further research with longitudinal cohorts to clarify the directionality and temporal ambiguity of these relationships is warranted to better support causal interpretation. The present study is also susceptible to Neyman (prevalence-incidence) bias [36], as it is a referral-based, cross-sectional analysis restricted to non-hospitalised individuals. This means that those who were not referred, had more severe acute infection, recovered quickly, or were unable to attend due to symptom burden are not represented. Following all patients from the point of COVID-19 diagnosis, irrespective of severity, would mitigate this and allow for identification of incident cases. However, the aim of this study was to specifically address the under-studied non-hospitalised individuals with persistent symptoms. Further, as respiratory symptoms were a pre-requisite for referral to the clinic and therefore study inclusion, the study is inherently subject to selection bias and the prevalences of breathlessness and dysregulated breathing cannot be directly compared with other cohorts. Indeed, the present study serves to highlight associated risk factors rather than provide prevalence data for these outcomes post-COVID. Another limitation is that cut-offs for outcome measures were not derived from post-COVID populations, meaning their sensitivity/specificity may not perfectly translate to participants included in the present study. With that said, using commonly adopted, pre-specified thresholds for the included PROMs and functional measures allows findings to be more consistently interpreted across studies. Similarly, although the Fried Frailty Index has only been formally validated for use in older adults (≥65 years), studies in middle-aged populations support its use in identifying frailty-associated morbidity and mortality risk [37], indicating prognostic utility. However, physical frailty is heterogeneous and different combinations of criteria are associated with different trajectories of disability and mortality [38]. In the context of post-COVID illness, some criteria may be disproportionately represented (e.g., exhaustion as a result of breathlessness), making it unclear how closely previously documented frailty-outcome associations can be mapped onto the population studied here. Additionally, given this was a referral-based cohort comprising predominantly women and middle-aged, White individuals, the generalisability of the findings may be limited. Larger, more diverse samples are needed to identify potential ethnicity-related disparities in COVID-19 sequelae. In the present study, ethnicity could not be categorised beyond White versus non-White due to limited data, hindering our insight. Complete-case analysis was used for each regression model. However, because missingness may have been influenced by unmeasured factors (i.e., not missing at random), the effect estimates reported may be biased and should be interpreted with caution. With that said, based on analysis of missingness, we do not expect complete-case analysis to unduly bias results. As PROMs were analysed in separate models, we could not disentangle the independent contributions of correlated variables (e.g., anxiety vs. depression). Future studies using larger cohorts should examine shared and unique contributions of the potentially relevant modifiable risk factors identified in the present analysis, to better inform intervention targets. Given the multiple outcomes and predictors examined, the risk of type I error is increased. However, as this analysis was exploratory and intended to identify potentially modifiable factors for future study, we did not adjust for multiple comparisons to avoid obscuring potentially meaningful signals. Notwithstanding these limitations, the potential for confounding and other biases were either reduced or explored by adjustment for key sociodemographic variables, assessment of missingness patterns, and sensitivity analyses.

## Conclusions

To summarise, among non-hospitalised patients referred to a respiratory clinic after COVID-19, women and individuals experiencing psychological distress and fatigue were disproportionately affected by post-acute sequelae. The fact that many participants had been experiencing symptoms for over a year stresses the need for earlier identification of symptoms, so that evidence-based management strategies can be implemented without delay. The findings suggest that multi-faceted, personalised rehabilitation strategies – including breathing control, graded exercise therapy and psychological support – should be considered to optimise recovery.

## Supporting information

S1 Table Number of individuals with missing data for variables included in regression models. (PDF)

S2 Table Pairwise overlap of outcomes. (PDF)

S1 Fig Participant flow chart. (PDF)

S2 Fig UpSet plot illustrating exclusive combinations of all outcomes. (PDF)

S3 Fig Spearman’s rank correlation heatmap for PROMs and SPPB. (PDF)

S1 Text Missing data analysis. (PDF)

S2 Text Packages used for regression analysis and reporting. (PDF)
