# Supplementary material for: The Nottingham recovery from COVID-19 research platform (NoRCoRP): Functional, clinical and patient-reported outcomes in adults referred to a post-COVID respiratory service
Source: PLoS One. 2026 Mar 5;21(3):e0344210. doi: 10.1371/journal.pone.0344210 (PMC12962452; doi:10.1371/journal.pone.0344210)
Supplement: S1 Table — (PDF) [file pone.0344210.s001.pdf]

S1 Table. Number of individuals with missing data for variables included in regression models for the 210 participants included in the main analytical sample.

Variable	N missing (%)
**Key confounders**	
Age	0 (%)
Sex	0 (%)
BMI	16 (8%)
Ethnicity	3 (1%)
Smoking	3 (1%)
IMD quintile	5 (2%)
**Exposure/outcome variables**	
Chalder Fatigue Score	34 (16%)
HADS anxiety/depression domain	34 (16%)
MRC dyspnoea score	0 (%)
Nijmegen score	15 (7%)
SPPB score	40 (19%)
EQ-5D VAS	36 (17%)
