# Supplementary material for: The Nottingham recovery from COVID-19 research platform (NoRCoRP): Functional, clinical and patient-reported outcomes in adults referred to a post-COVID respiratory service
Source: PLoS One. 2026 Mar 5;21(3):e0344210. doi: 10.1371/journal.pone.0344210 (PMC12962452; doi:10.1371/journal.pone.0344210)
Supplement: S2 Table — (PDF) [file pone.0344210.s002.pdf]

S2 Table. Pairwise overlap of outcomes in those with complete outcome data (n = 148). Percentages are row-wise: each cell shows overlap count/number with row outcome.

	MRC ≥3	NQ >23	Low SPPB	Fatigue	Anxiety	Depression
**MRC ≥3** (n=86/148, 56%)	-	68 (79%)	35 (41%)	81 (94%)	54 (63%)	51 (59%)
**NQ >23** (n=97/148, 66%)	-	-	40 (41%)	94 (97%)	71 (73%)	60 (62%)
**Low SPPB** (n=48/148, 32%)	-	-	-	47 (98%)	35 (73%)	32 (67%)
**Fatigue** (n=133/148, 90%)	-	-	-	-	90 (68%)	71 (53%)
**Anxiety** (n=93/148, 63%)	-	-	-	-	-	62 (67%)
**Depression** (n=74/148, 50%)	-	-	-	-	-	-

These data are descriptive only and not indicative of associations.
