# Supplementary material for: The Nottingham recovery from COVID-19 research platform (NoRCoRP): Functional, clinical and patient-reported outcomes in adults referred to a post-COVID respiratory service
Source: PLoS One. 2026 Mar 5;21(3):e0344210. doi: 10.1371/journal.pone.0344210 (PMC12962452; doi:10.1371/journal.pone.0344210)
Supplement: S1 Fig — (PDF) [file pone.0344210.s003.pdf]

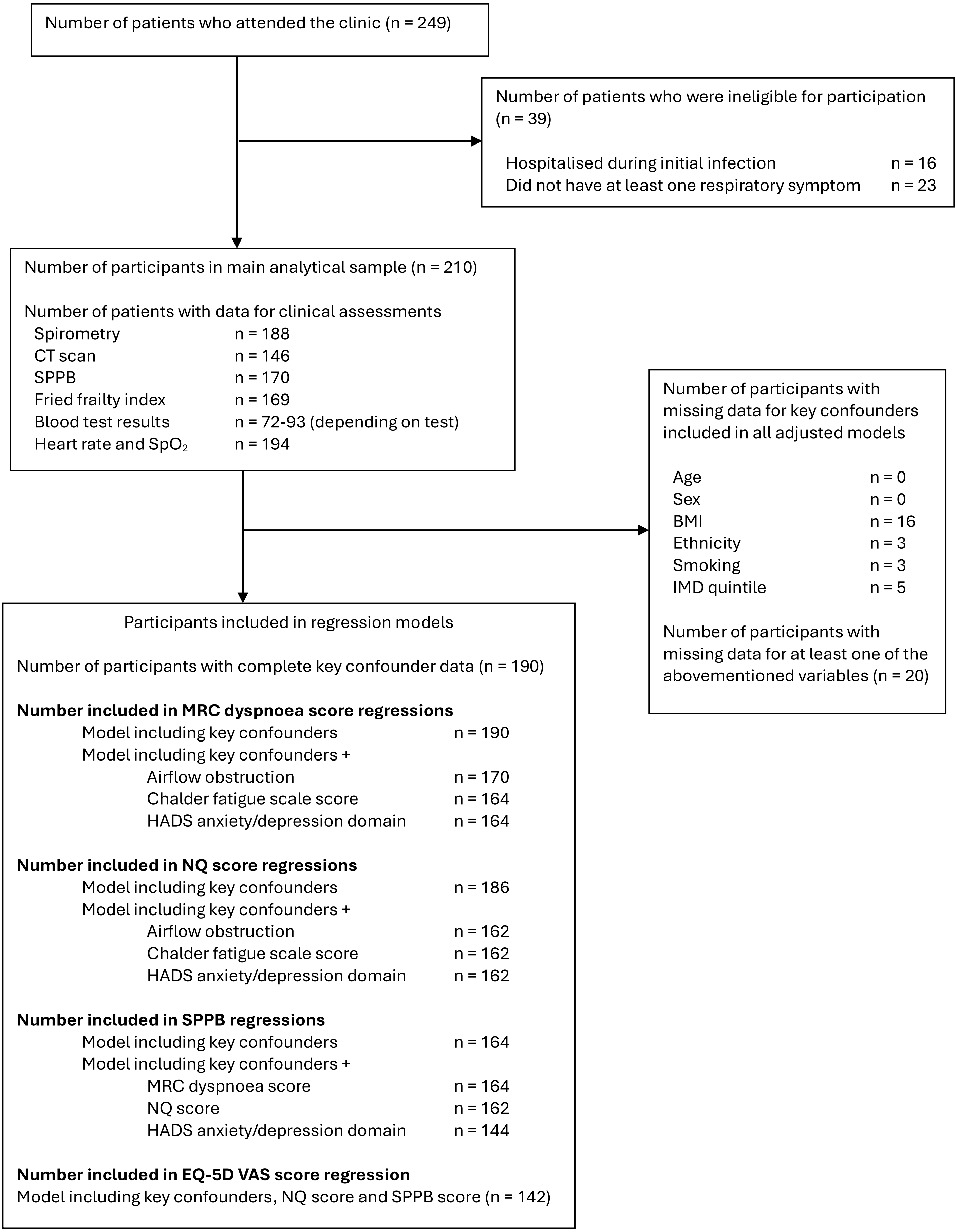

S1 Figure. Participant flow chart

IMD, Index of Multiple Deprivation; MRC, Medical Research Council; HADS, Hospital Anxiety and Depression Scale; NQ, Nijmegen Questionnaire; SPPB, Short Physical Performance Battery; EQ-5D VAS, EuroQol-5 Dimensions Visual Analogue Scale; SpO_2, Resting peripheral oxygen saturation.
