# Supplementary material for: The Nottingham recovery from COVID-19 research platform (NoRCoRP): Functional, clinical and patient-reported outcomes in adults referred to a post-COVID respiratory service
Source: PLoS One. 2026 Mar 5;21(3):e0344210. doi: 10.1371/journal.pone.0344210 (PMC12962452; doi:10.1371/journal.pone.0344210)
Supplement: S2 Fig — (PDF) [file pone.0344210.s004.pdf]

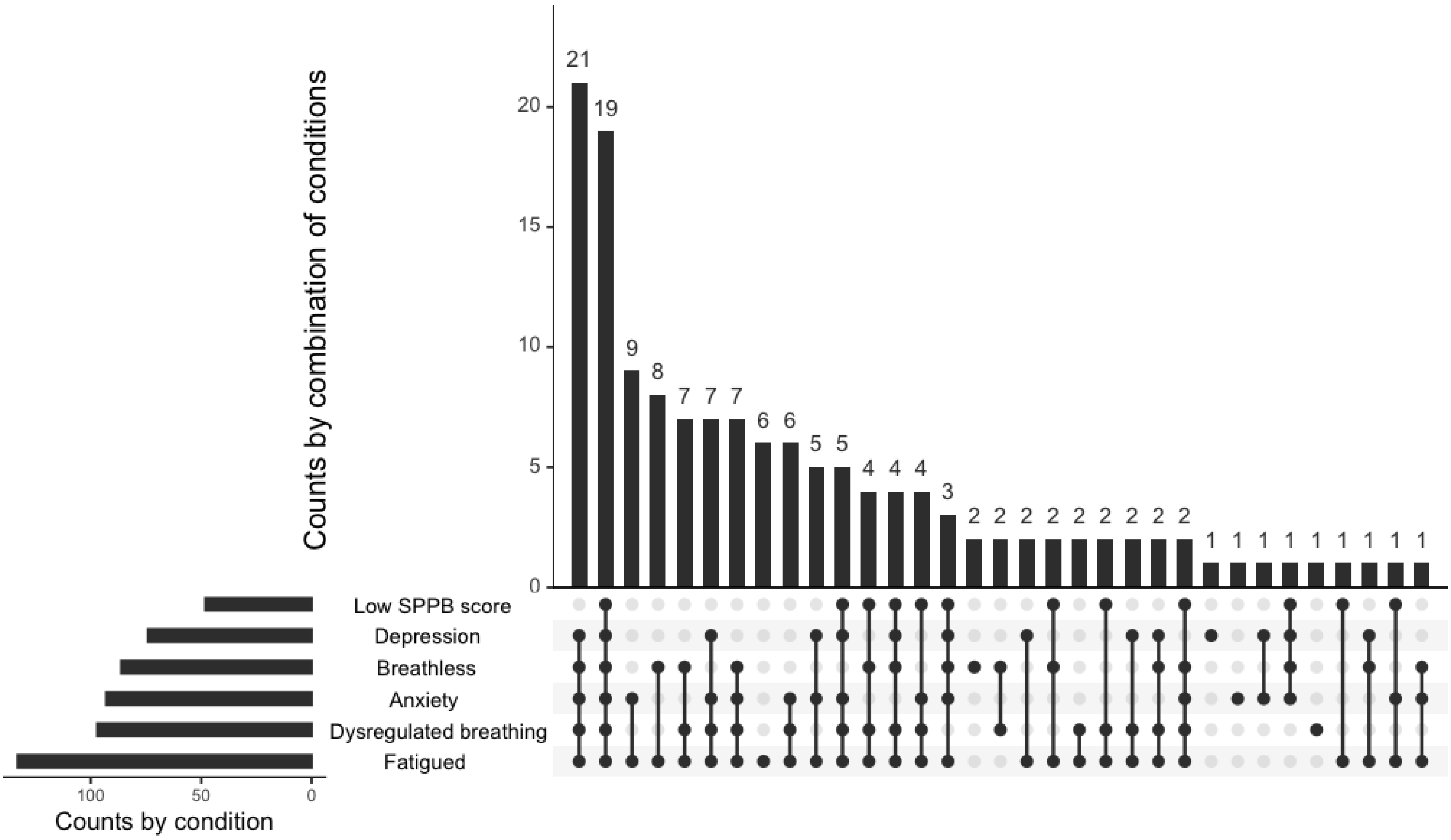


**S2 Figure.** UpSet plot illustrating exclusive combinations of outcomes and the number of participants in each (n total = 142). Combinations are indicated by the dot matrix (bottom right) with the corresponding participant counts shown by the bars above. Total counts for each condition are shown by the bars on the left. Six participants had no outcomes and are not included in the figure.
