# Supplementary material for: The Nottingham recovery from COVID-19 research platform (NoRCoRP): Functional, clinical and patient-reported outcomes in adults referred to a post-COVID respiratory service
Source: PLoS One. 2026 Mar 5;21(3):e0344210. doi: 10.1371/journal.pone.0344210 (PMC12962452; doi:10.1371/journal.pone.0344210)
Supplement: S3 Fig — (PDF) [file pone.0344210.s005.pdf]

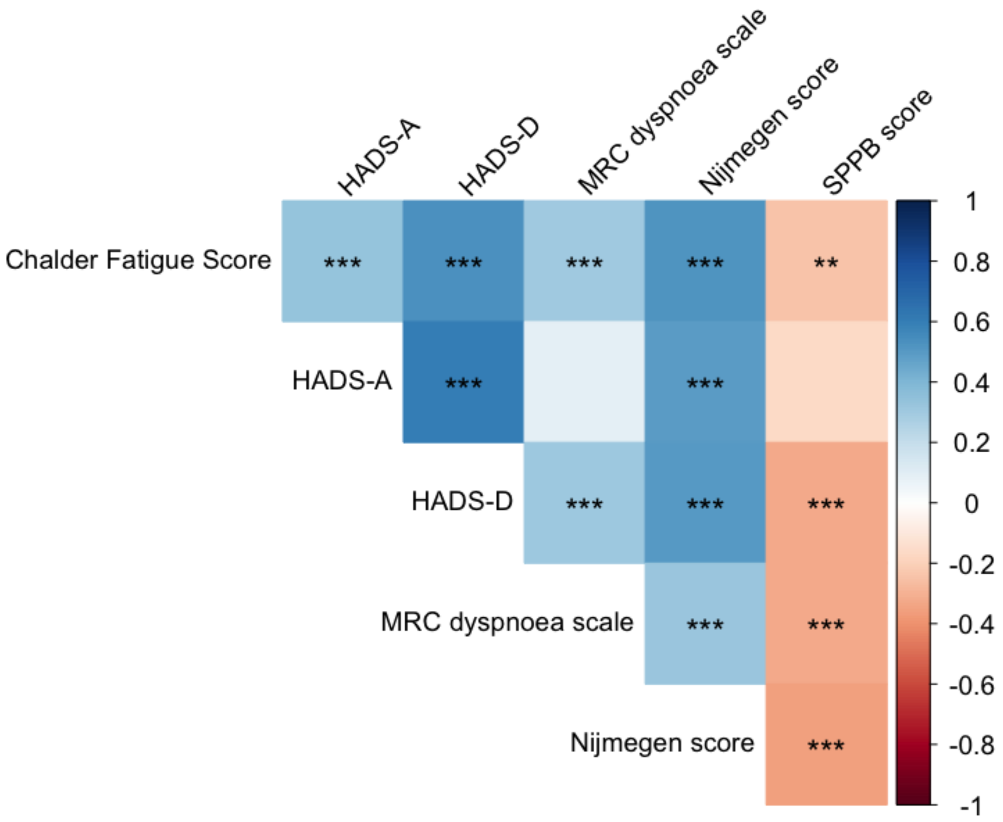

S3 Figure. Spearman's rank correlation heatmap for PROMs and SPPB (n = 148).

* p < 0.05, ** p < 0.01, *** p < 0.001

HADS, Hospital Anxiety and Depression Score; MRC, Medical Research Council; SPPB, Short Physical Performance Battery; PROMs, patient reported outcome measures.
