# Supplementary material for: The Nottingham recovery from COVID-19 research platform (NoRCoRP): Functional, clinical and patient-reported outcomes in adults referred to a post-COVID respiratory service
Source: PLoS One. 2026 Mar 5;21(3):e0344210. doi: 10.1371/journal.pone.0344210 (PMC12962452; doi:10.1371/journal.pone.0344210)
Supplement: S1 Text — (PDF) [file pone.0344210.s006.pdf]

S1 Text. Missing data analysis

The number of participants with missing data for variables included in regression models is presented in Supplementary Table 1. Missing data were minimal for most key confounders, with BMI having the highest proportion at 8%. Exposures/outcomes had greater missingness (SPPB: 19%, EQ-5D VAS: 17%, Chalder Fatigue Scale and HADS: 16%, NQ score: 7%). To explore patterns of missingness, binary indicators for each exposure/outcome variable (1 = missing, 0 = observed) were created and used to model their associations with key demographic factors (age, sex, ethnicity, BMI, smoking status, and IMD quintile) using logistic regression. Only younger age was significantly associated with missingness for the Chalder Fatigue Scale and HADS (OR per year increase = 0.96, 95% CI = 0.93 to 0.99) and EQ-5D VAS (0.96, 0.94 to 0.99). Evidently, there may be limited availability of strong predictors to support a robust multiple imputation model. In addition, some missingness is likely to be influenced by unmeasured contextual factors (e.g., differential engagement in testing related to symptom burden) that are difficult to account for, meaning that the assumption of missing at random may not hold. For these reasons, a complete-case approach was adopted for our analyses.
