# Supplementary material for: The Nottingham recovery from COVID-19 research platform (NoRCoRP): Functional, clinical and patient-reported outcomes in adults referred to a post-COVID respiratory service
Source: PLoS One. 2026 Mar 5;21(3):e0344210. doi: 10.1371/journal.pone.0344210 (PMC12962452; doi:10.1371/journal.pone.0344210)
Supplement: S2 Text — (PDF) [file pone.0344210.s007.pdf]

S2 Text. Packages used for regression analysis and reporting.

- **Base R:** primary model fitting for linear and logistic regression (e.g., lm, glm, AIC)
- **rms**: regression modelling with restricted cubic splines to test for non-linear associations
- **lmtest:** likelihood ratio tests (lrtest) for comparing models (linear vs. non-linear)
- **car:** multicollinearity assessment for variance inflation factors (vif)
- **performance:** good-of-fit checks (e.g., Hosmer-Lemeshow)
- **emmeans:** estimated marginal means and pairwise comparisons
- **broom:** ‘tidy’ regression outputs for reporting
